# PReS-FINAL-2035: Fatty acid profiling: potential new biomarkers in JIA

**DOI:** 10.1186/1546-0096-11-S2-P48

**Published:** 2013-12-05

**Authors:** C Boros, WT CHAM, J Fletcher, E Ranieri

**Affiliations:** 1 Uni Dept Paedaitrics, Womens and Childrens Hospital, Australia; 2 Biochemical genetics, IMVS pathology, Adelaide, Australia

## Introduction

The prostanoids are a family of biologically active lipids derived from the 20-carbon essential fatty acids (LCPUFA) which are involved in all aspects of the immune response including the resolution of inflammation. ω3-fatty acids, EPA DPA and DHA are anti-inflammatory, whilst the ω6-fatty acid, Arachidonic acid (AA) and its metabolites: 13(S)-HETE, TXB2, PGF2α and 6-k-PGF1α are pro-inflammatory. Liquid Chromatography Tandem Mass Spectrometry (LC-MSMS) allows analyses of multiple prostanoids with high accuracy using 3 mm blood spots. This method has never been used in JIA and may find biomarkers which can help predict disease activity and treatment response.

## Objectives

To measure prostanoid profiles in patients with JIA using LC-MSMS.

## Methods

254 samples from 114 JIA patients and 6 healthy controls (HC) were collected onto specially prepared filter papers and analysed using LC-MSMS.

## Results

The JIA M:F ratio was 1:1.4, the average age at study entry (9.4 ± 5.0 y), average disease duration (56.1 ± 46.1 m), with 25% JIA receiving treatment with NSAID, 11% with Methotrexate (MTX), and 10% with Biologics. 13(S)HODE and DHA levels were significantly different between JIA patient groups (p = 0.05 for both; 13(S)HODE oligo vs poly p = 0.02; DHA SoJIA vs RF+ Poly p = 0.007). There was a positive correlation between JADAS and PGB2 (p = 0.046). There were lower levels of pro-inflammatory prostanoids in JIA (Table 1).

**Table 1:** 

Prostanoid/JIA subtype	Oligo	Extended Oligo	Poly	RF+ Poly	JPSA	ERA	SoJIA
HODE			p = 0.001		p = 0.002	p = 0.01	

DPA			p = 0.02		p = 0.017	p = 0.01	

PGF2a	p = 0.001	p = 0.03	p = 0.007				p = 0.04

TBX2	p = 0.009	p = 0.004	p < 0.001	p = 0.007	p = 0.04		

## Conclusion

In our JIA cohort, we found that PGB2 is correlated with disease activity and that levels of pro-inflammatory prostanoids are reduced, particularly in polyarthritis. This may reflect the degree to which the pro-resolving prostanoids are activated in patients with relatively long average disease duration. It is also possible that measurement of a combination of prostanoids will help us predict changes in disease activity and treatment response over time more accurately. Longitudinal analysis the relationship between disease activity and prostanoid profiles is underway.

## Disclosure of interest

None declared.

